# Green seaweeds fatty acids and heterocyclic derivatives against cancer: Opinion on future nutraceutical application

**DOI:** 10.3389/fonc.2023.1145919

**Published:** 2023-02-14

**Authors:** Nurmeilita Taher, Feny Mentang, Roike Iwan Montolalu, William Ben Gunawan, Nurpudji Astuti Taslim, Nelly Mayulu, Fahrul Nurkolis

**Affiliations:** ^1^ Fishery Products Technology Study Program, Faculty of Fisheries and Marine Sciences, Sam Ratulangi University, Manado, North Sulawesi, Indonesia; ^2^ Alumnus of Nutrition Science Department, Faculty of Medicine, Diponegoro University, Semarang, Central Java, Indonesia; ^3^ Clinical Nutrition, Faculty of Medicine, Hasanuddin University, Makassar, South Sulawesi, Indonesia; ^4^ Food and Nutrition, Universitas Muhammadiyah Manado, Manado, North Sulawesi, Indonesia; ^5^ Biological Sciences, State Islamic University of Sunan Kalijaga (UIN Sunan Kalijaga), Yogyakarta, Yogyakarta, Indonesia

**Keywords:** green seaweed, fatty acid, heterocyclic derivatives, cancer, nutraceutical, functional food

## 1 Introduction

One of the most significant issues endangering human health today is cancer which has a direct correlation with mortality (1). Global cancer data indicate that there were 10.0 million cancer deaths (9.9 million excluding nonmelanoma skin cancer) and 19.3 million new cases globally in 2020 (2). Depending on the features and stage of the tumor, the treatment of cancer often entails a mix of treatments, such as surgery, radiotherapy, chemotherapy, and most recently, immunotherapy (3). As a result, the creation of medications targeting a particular cancer-related target in conjunction with a thorough comprehension of how pharmaceuticals interact with the biology of human tumors has emerged as the key to the present quest to cure cancer (4). Despite great efforts of research, effective cancer treatment is still lacking, implying the need for other sources of the cancer-treating agent such as natural sources. Natural sources – especially underutilized marine products – have promising potential as functional food or nutraceutical with anticancer properties (5, 6).

Seaweed – which is frequently consumed in East Asian nations – provides nutrients such as minerals, vitamins, soluble dietary fibers, and flavonoids that are thought to protect against illnesses brought on by a sedentary lifestyle (7). The notion of using seaweed as functional foods or nutraceuticals with nutritional advantages beyond their basic macronutrient content is now a focus of various studies and developments. Furthermore, several studies highlighted that dietary seaweed intake is a protective factor against cancer by decreasing the risk and mortality of cancers (8, 9). Due to the anticarcinogenic properties of its bioactive compounds, which include preventing or delaying the development of cancer in both *in vitro* and *in vivo* models as well as regulating tumor cell metabolism, cell proliferation, apoptosis, and the cell cycle, dietary seaweed consumption has recently attracted attention on a global scale (10–12).

Green seaweeds are good sources of monocarboxylic acids with a significant amount of hydrocarbon chain which are known as fatty acids (FA). They may generally be classified as either saturated or unsaturated and are produced by the cleavage of natural fats and oils like triacylglycerols or phospholipids (13). Moreover, the role of heterocyclic derivatives in improving the cytotoxicity activity of cancer drugs and therapies has been highlighted (13). There has been no publication of opinion papers discussing the combination of green seaweed’s fatty acids and heterocyclic derivatives as anticancer nutraceuticals. This opinion aims to interpret green seaweeds’ potential as anticancer nutraceuticals based on their fatty acids content and their potential application when incorporated into a fatty acid-heterocyclic hybrid (FAHH).

## 2 Green seaweeds and anticancer properties

The marine, photosynthetic algae known as seaweed are abundant in all oceans and categorized into three primary classifications – Phaeophyceae (brown algae), Rhodophyta (red algae), and Chlorophyta (green algae) (14). Diverse settings, such as tidal or subtidal areas, or shallow coastal waters, are home to marine algae. Based on the existence of photosynthetic pigments, green algae are members of the Chlorophyta phylum of the Plantae kingdom (15). As stated above, seaweed has several health-benefiting potentials (16–18). Numerous sulfated and carboxylated polysaccharides, including alginate, ulvan, and fucoidan, as well as carotenoids, phenolic compounds, tocopherols, and peptides, are among the many bioactive substances found in seaweed (19).

Seaweeds have high carbohydrate content (31.8% to 59.1%) and ash content (12.4% to 29.9%) and a low total lipid content that varies from 0.60% to 7.87% (20–23). Different seaweed lipids have a wide range of fatty acid compositions, with saturates making up 51.9–67.4%, monoenes 22.0–32.9%, and polyunsaturated fatty acids (PUFA) 9.2–19.1% (22). Docosahexaenoic and Eicosapentaenoic acids are two examples of the omega-3 (n-3) fatty acids that make up most of the lipids in algae, while the two monounsaturated omega-6 (n-6) fatty acids that are most frequently found in algae are linoleic and arachidonic acids (24). Seaweeds also contained palmitic and myristic (fundamental saturated fatty acids) and phytanic acids (branched chain fatty acids) (20, 21). The fatty acid content of several green seaweeds were presented in **Table 1**.

**Table 1:** Content of fatty acids in several green seaweeds.

	Total Lipid	Total Fatty Acid	Total ω-3	Total ω-6	ω-6/ω-3 Ratio	References
*Ulva linza*	3.20 – 4.14% of DW	71.25 – 89.73 of TL				(20)
*Cladophora patentiramea*	9.87 ± 0.43% of DW	3.46% of DW	0.26% of DW	0.99% of DW	3.81	(25)
*Caulerpa sertularioides*	13.04 ± 1.46% of DW 1.63 ± 0.21% of DW	2.76 ± 0.49% of DW	0.71 ± 0.13% of DW 0.96%	0.36 ± 0.08% of DW 12.99%	0.51	(25, 26)
*Derbesia tenuissima*	12.14 ± 0.59% of DW 15.2 ± 1.5% of DW	3.96 ± 0.51% of DW 5.1 ± 0.7% of DW 5.07 ± 0.12% of DW	1.22 ± 0.46% of DW 1.9 ± 0.1% of DW 3.80 ± 0.05% of DW	0.60 ± 0.03% of DW 1.24 ± 0.02% of DW	0.49 0.33 ± 0.01	(25, 27)
*Ulva ohnoi*	2.3 ± 0.3% of DW	2.0 ± 0.0% of DW 2.04 ± 0.03% of DW	0.9 ± 0.0% of DW 4.22 ± 0.06% of DW	0.27 ± 0.01% of DW	0.06 ± 0.00	(27)
*Ulva lactuca*	3.6 ± 0.42% of DW 1.43 ± 0.17% of DW 1.37 ± 0.04% of DW 1.48 ± 0.05% of DW		4.8 ± 0.83% of TFA 0.98 – 1.07% 14.1 ± 0.0% of TFA	5.1 ± 1.14% of TFA 13.69 – 14.67% 12.1 ± 0.3% of TFA	1.06 0.86	(22, 26, 28, 29)
*Enteromorpha intestinalis*	2.9 ± 0.25% of DW		9.8 ± 0.60% of TFA	4.8 ± 0.75% of TFA	0.49	(22)
*Codium tomentosum*	3.6 ± 0.2% of DW	2.76 ± 0.02% of DW	31.57 ± 0.72% of TL	10.99 ± 0.19% of TL	0.35 ± 0.02	(21)
*Caulerpa lentillifera*	1.40 ± 0.08% of DW					(28, 30)
*Halimeda opuntia*	0.18 ± 0.06% of DW					(28)
*Ulva armoricana*	2.62 ± 0.04% of DW		23.9 ± 0.1% of TFA	3.7 ± 0.1% of TFA	0.15	(31)
*Caulerpa racemosa*	4.5% of DW 3.73 ± 0.19% of DW 6.70 ± 0.11% of DW		0.96 – 1.12%	10.65 – 10.96%	0.97 – 7.54	(26, 32)
*Ulva fasciata*	4.1% of DW				1.39 – 120.40	(32)
*Rhizoclonium riparium*			20.1 ± 0.1% of TFA	12.0 ± 0.5% of TFA	0.60	(29)
*Ulva prolifera*			14.0 ± 0.3% of TFA	24.7 ± 0.8% of TFA	1.76	(29)
*Ulva intestinalis*			14.6 ± 0.4% of TFA	10.7 ± 0.0% of TFA	0.73	(29)
*Chaetomorpha linum*			8.9 ± 0.2% of TFA	2.4 ± 0.1% of TFA	0.27	(29)

DW, Dry Weight; TFA, Total Fatty Acid; TL, Total Lipid (Fat).

Green seaweeds are member of algae which also known as Chlorophyta. They are less common than brown and red algae and exhibit greenish-yellow to dark green coloration (23). Over 900 types of green seaweed, over 1500 species of brown seaweed, and over 4000 species of red seaweed have been identified globally. While green and red seaweed only grows in tropical environments, brown seaweed may be found in temperate areas (33). In general, these three types of seaweed have different coloring pigments and compositions of the cell wall’s polysaccharides (34). The cell wall polysaccharides, which account for 38–54% of the dry algal matter in green seaweeds, are interesting bioactive compounds that can be used for various applications. Green algae contain water-soluble sulfated polysaccharides that have antioxidant and anticancer properties (35). On the other hand, the proximate and mineral compositions of green seaweed have been determined (19, 28, 36), and compared to brown seaweed, green seaweed has lower protein, lipid, and zinc content with higher carbohydrate content (19). A review by Peñalver et al., 2020 also pointed out that overall, green seaweed has lower protein, lipid, and ash content in comparison to red and brown seaweed (23). The major active compounds of green seaweed are flavonoid compounds, steroids, triterpenoids, saponins, alkaloids, and phenol hydroquinone, which are supplemented with the ability as antioxidants (28).

## 3 Anticancer properties of fatty acids and heterocyclic compounds

**Figure 1:**
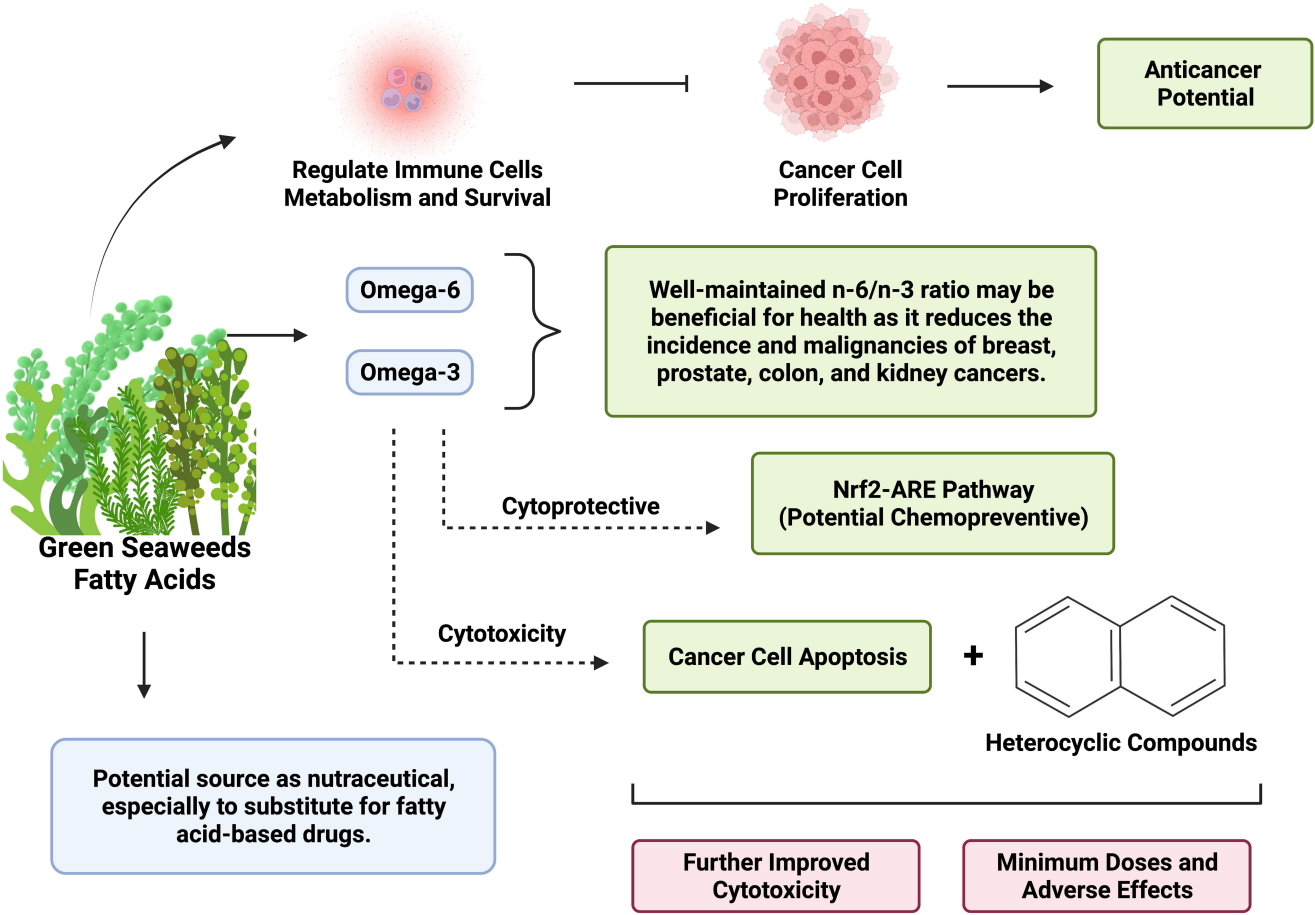
Big picture of the anticancer potential of fatty acid-heterocyclic hybrid of green seaweeds as nutraceuticals. Created with BioRender.com premium license by Fahrul Nurkolis.

Elevated risk for several types of human cancer has been linked to having an excessive quantity of serum free fatty acid level. By controlling fatty acid metabolism, cancer cells can reroute metabolic pathways to fulfill energy needs (**Figure 1**). Energy, macromolecules for membrane synthesis, and lipid signals are all crucial components of reprogramming in fatty acid metabolism during the development of cancer (37). Given the significance of both fatty acid production and oxidation in cancer cells, it is possible to target fatty acid metabolism for anticancer therapy by using pharmacological inhibition to reduce cell division, growth, and transformation (38). Furthermore, promising methods to work in conjunction with immunotherapies have emerged, especially involving controlling fatty acids metabolism in immune cells, as primary immune cells require fatty acids metabolism to survive and perform at their best (39). The activation of fatty acid oxidation (which limit the availability of fatty acid) is linked with lower cancer cell proliferation and improved cancer outcomes (40, 41).

Some of the trending fatty acids are the omega groups (n-3, n-6, and n-9). Through selective cytotoxicity, Omega-3 polyunsaturated fatty acids (n-3 PUFAs; Ω3 fatty acids) can cause cancer cells to undergo apoptosis (42). Subsequently, n-3 PUFAs could make tumor cells more susceptible to conventional treatments, which might boost their effectiveness. Omega-9 (n-9) has also been studied for its antiproliferative activity against cancer by suppressing the migration and stimulation of tumor cells (43). If the ratio of n-6 to n-3 is taken into account in functional foods and nutraceuticals, these PUFAs may be advantageous for human health (31). An earlier investigation found a reduction in the incidence of breast, prostate, colon, and kidney malignancies with an n-6/n-3 fatty acid ratio lower than 2/1 to 4/1 (44). In this sense, green seaweeds were proven to prevent cancer due to their n-6/n-3 fatty acid ratio being 0.5 to 1.1 (22). *Ulva lactuca*, a green alga, produces unsaturated fatty acid components that are cytoprotective Nrf2-ARE pathway activators, demonstrating their potential as chemopreventive dietary unsaturated fatty acids (45).

Heterocycles are defined as “cyclic compounds possessing ring members atoms of at least two other elements” (46). Heterocycles are essentially made of elements other than carbon, with oxygen, nitrogen, and sulfur being the most common substituents. Heterocycles have become parts of cancer chemotherapy drugs with interesting cytotoxicity profiles, especially nitrogen-containing heterocyclic compounds such as pyrrole, purine, pyrrolidine, pyridine, imidazole, pyrimidines, pyrazole, indole, quinoline, oxadiazole, azole, benzimidazole (47, 48). Using a fatty acid-heterocyclic hybrid brings many beneficial influences on cancer therapies. For therapy agents that have cytotoxicity activity, FAHH will improve their therapeutic efficacy, preventing high-dose drugs which can lead to toxicity (13). The activation of fatty acids has significant roles to play in carcinogenesis and cancer development (49), such as cancer immunotherapy efficacy boosters (50). This activation may be influenced by the addition of heterocyclic compounds (e.g. through condensation reactions) which alter the systems and structures of fatty acids (13). More interestingly, FAHH – as a hybrid molecule – may also have additional antimicrobial, antifungal, and antituberculosis activities aside from anticancer (51).

## 4 Future implication and direction in nutraceutical application

The main idea of green seaweeds as nutraceutical with anticancer potential in terms of fatty acid-heterocyclic hybrid was presented in **Figure 1**. Taking into account the various health-benefiting potentials of green seaweeds, these algae can be continuously developed into functional foods and nutraceuticals, such as through fortification and incorporation of green seaweeds into new food or existing ones. In general, green seaweeds have alkaloids, phenols, flavonoids, quinine, and polysaccharides that exhibit anticancer activity and provide antioxidant-associated health benefits (5). On the other hand, food fermentation has been reported capable to increase the bioactive compounds and antioxidants of food, further improving the health benefits contained in the food product (52). Combined with the fatty acids profile of green seaweeds, greater anticancer potentials can be expected from green seaweed-based food. Furthermore, there is a growing interest in the application of FAHH as an anticancer agent. This trend highlights the hidden “treasure” of marine products especially since fatty acids are well-contained in green seaweeds and heterocyclic compounds can also be found in marine products (53, 54). The development and incorporation of fatty acid-heterocyclic hybrid through micro- and nanoencapsulation technologies will be crucial in the discovery of novel nutraceuticals, accompanied by greater value of stability and bioaccessibility.

Green seaweed has fatty acid content that makes up most of its fat content. Fatty acids have displayed a significant influence on the prevention and management of cancer. Recent evidence and insight regarding the incorporation of heterocyclic compounds into fatty acids are proposed to increase the anticancer cytotoxicity and efficacy of cancer therapeutic agents, along with other additional health-benefiting properties. Therefore, green seaweeds showcase their potential as anticancer nutraceuticals, and deeper research on this topic is crucial and greatly encouraged.

## Author contributions

NT, FM, RM, WG, NT, NM, and FN: Contributed to the conceptualization with the design of the critical opinion study, drafted the manuscript, edited-revised it, and approved the final version of the submitted manuscript. All authors contributed to the article and approved the submitted version.
